# PARP Inhibitors in Pancreatic Cancer: From Phase I to Plenary Session

**DOI:** 10.17140/POJ-3-e011

**Published:** 2019-12-20

**Authors:** Rajvi Patel, Daniel Fein, Carolina B. Ramirez, Kevin Do, Muhammad W. Saif

**Affiliations:** 1 Northwell Health Cancer Institute and Donald and Barbara Zucker School of Medicine, Hofstra/Northwell, NY, USA; 2 Tufts University, School of Medicine, Tufts Cancer Center, Boston, MA, USA

**Keywords:** Pancreatic cancer, Chemoresistance, DNA damage repair, Synthetic lethality, *BRCA1/2*, Germline mutations, Genomics

## Abstract

Survival rates for pancreatic cancer remain dismal. Current standard of care treatment regimens provide transient clinical benefit but eventually chemoresistance develops. Tumors deficient in deoxyribonucleic acid (DNA) damage repair mechanisms such as *BRCA* mutants show better responses to platinum based agents, however, such tumors can utilize the poly(adenosine diphosphate [ADP]–ribose) polymerase (PARP) pathway as a salvage mechanism. Therefore, inhibition of PARP pathway could lead to tumor destruction and synthetic lethality in presence of *BRCA* mutation. Various PARP inhibitors have been approved for treatment of patients with germline or somatic *BRCA* mutant breast and ovarian cancer. This provides basis of using PARP inhibitors in patients with pancreatic cancer that harbor *BRCA* mutation. A recent phase III Pancreas Cancer Olaparib Ongoing (POLO) study showed impressive results with near doubling of progression free survival compared to placebo (7.4 *vs* 3.8 months). These results highlight the importance of germline testing for all patients with pancreatic cancer and inclusion of additional deficiencies in homologous recombination repair (*ATM* and *PALB2*) including *BRCA* variants of uncertain significance should be further explored.

The fatality of pancreatic ductal adenocarcinoma (PDAC) continues to rise regardless of current efforts to improve survival. Given the subtle clinical presentation, most patients have advanced disease at the time of diagnosis. Hence, the 5-year-survival rate is 3% and the median overall survival (OS) is around 6-months for patients with metastatic disease.^1^ Currently, there are limited treatment options that include combination regimens with oxaliplatin, irinotecan, fluorouracil and leucovorin (FOLFIRINOX) or gemcitabine plus nab-paclitaxel.^2,3^ However, chemotherapy provides a transient clinical benefit and eventually PDAC becomes resistant to conventional therapies.

Mechanisms for chemotherapy resistance may be related to tumor microenvironment or intrinsic genetic alterations. A proposed mechanism for development of PDAC includes multiple steps where premalignant lesions called pancreatic intraepithelial neoplasia (PanIN), intraductal papillary mucinous neoplasm (IPMN), and mucinous cystic neoplasm (MCN) develop into carcinoma. *KRAS* mutation is crucial for pancreatic carcinogenesis and more than 90% of pancreatic tumors express KRAS mutated protein.^4–7^ Inactivation of cyclin-dependent kinase inhibitor 2A (*CDKN2A*), *SMAD4*, *TP53*, and other tumor suppressor genes are also key elements implicated in this progression model.

Additionally, genes involved in deoxyribonucleic acid (DNA) damage repair (DDR) may also contribute to the pathogenesis of PDAC and deficiency in DDR mechanism leads to an increased risk of cancer. It is known that poly(adenosine diphosphate [ADP]–ribose) polymerase (PARP) 1/2 detect DNA damage and promote its repair.^8^ Importantly, studies have demonstrated the clinical benefit of PARP inhibition in carriers of *BRCA1* or *BRCA2* mutation.^9^

Tumors that harbor mutations in genes related to double strand DNA repair such as *BRCA1*, *BRCA2*, *PALB2*, or *ATM* have been associated to have better responses to platinum based chemotherapy agents. This is explained by the fact that platinum compounds generate double-strand DNA breaks that cannot be repaired due to mutation in double-strand DNA repair genes. However, tumors that harbor mutations in homologous recombination genes, can utilize the Poly (ADP-ribose) polymerase (PARP) pathway that is involved in single strand DNA break repair as a salvage mechanism to repair DNA damage. Therefore, inhibition of PARP mediated pathway could lead to tumor destruction and synthetic lethality in presence of *BRCA* mutations (Figure 1). Based on this, various PARP inhibitors have been approved for treatment of patients with germline or somatic *BRCA* mutant breast and ovarian cancer (Table 1).

A recent study of whole genome sequencing of 638 patients with familial pancreatic cancer showed mutations in the *BRCA2* gene accounted for the largest fraction of known familial pancreatic cancer genes and was found in 5–10% of the families.^10^ Among patients with no family history of PDAC, *BRCA2* mutation is found in 2% and *BRCA1* mutation is found in 1% of the patients or less.^11^ In the Ashkenazi Jewish population with PDAC, a much higher incidence of *BRCA* mutations are found and seen in up to 13.7% of unselected cases.^12^ Therefore, PARP inhibitors may play an important role in treatment of pancreatic cancer with germline or somatic *BRCA1*/*BRCA2* mutations as well.

## PARP INHIBITORS PHASE I STUDIES

Phase I studies were conducted after having demonstrated synthetic lethality with PARP inhibitors using *in vitro* models. In a study of 60 patients, 22(37%) of whom were known carriers of a *BRCA 1* or *BRCA 2* mutation, received olaparib at escalating doses. This study recruited patients 18-years of age or older with treatment refractory solid tumors. Dosing of olaparib was started at 10 mg daily for two of every three weeks, and was doubled every cycle of treatment, if tolerated. This trial demonstrated that the maximum tolerated dose of olaparib to be 400 mg twice daily. The most common side effects included grade 1–2 nausea, and fatigue in one third of patients. The most common grade 3–4 toxicities were lymphopenia and anemia, which occurred in up to 5% of the study population. Overall, 23 patients with *BRCA* mutation were treated and 19 patients were evaluated for response. 12 of 19 (63%) patients had clinical benefit defined as radiologic response (complete or partial response) based on response evaluation criteria in solid tumors (RECIST), tumor-marker responses defined as a decline in the tumor-marker level of more than 50% that was sustained for at least 4-weeks, or stable disease for a period of 4-months or more.^9^

## PARP INHIBITORS PHASE II STUDIES

Following promising phase I trial results, several phase II trials were conducted looking at PARP inhibitors in patients with germline *BRCA1* or *BRCA2* mutations. In a study of 298 patients with pre-treated solid tumors, 23 of whom had pancreatic cancer, patients received olaparib at 400 mg twice daily. The response rate was found to be 26% in all patients and 22% in patients with pancreatic specifically.^13^ Another multicenter phase II study, RUCAPANC, enrolled patients with *BRCA1* or *BRCA2* mutations and pancreatic cancer. In this study, 19 patients were enrolled and treated with the PARP inhibitor Rucaparib at 600 mg twice a day. Clinical responses were seen in 3/19 (16%) of the patients.^14^ Currently there are no head to head trials comparing various PARP inhibitors comparing efficacy. A meta-analysis presented at the Society of Gynecologic Oncology (SGO) meeting 2018 showed no difference in efficacy among the three PARP inhibitors but demonstrated a more favorable safety profile for olaparib, associated with a reduced odds ratio of grade 3 or greater adverse events and treatment interruption.^15^

## PARP INHIBITORS PHASE III STUDIES

The results of a recently published phase III Pancreas Cancer Olaparib Ongoing (POLO) trial were chosen for presentation at the plenary session of the American Society of Clinical Oncology (ASCO) 2019 meeting, highlighting the great excitement for the emerging roles of PARP inhibitor therapy in numerous solid tumors.^16^ Patients with metastatic pancreatic cancer enrolled for this randomized, double-blind, placebo-controlled phase III trial must have harbored a deleterious germline mutation of *BRCA1* or *BRCA2* (confirmed by central testing with the BRCAnalysis CDx test) and received at least 16-weeks of continuous first-line platinum-based chemotherapy for eligibility. Patients were randomly assigned in a 3:2 ratio to receive olaparib 300 mg twice daily or placebo (PBO) as a maintenance therapy started within 4–8 weeks after the final dose of first-line chemotherapy. Of 3315 patients screened for eligibility, 154 underwent randomization with 92 assigned to receive olaparib and 62 to receive placebo. The majority (>80% in each group) received a variant of FOLFIRINOX with an option to hold the platinum component after 16-weeks if toxicities arised. Although the duration of first-line chemotherapy was not limited, the majority in each group received therapy ranging from 16-weeks to 6-months (66% in olaparib, 65% in PBO).

The study met its primary endpoint impressively with near doubling of progression free survival (PFS) in the olaparib group compared to placebo (mPFS 7.4 *vs*. 3.8 mo, HR 0.53, 95% CI 0.35–0.82, *p*=0.004), generating an enthusiasm shown similar to the practice-changing SOLO1 olaparib maintenance trial for newly diagnosed advanced ovarian cancer (Table 2).^17^ Significant responses were seen in 20 patients in the olaparib group (20%) compared to 6 in PBO (10%), with 2 complete responses seen with olaparib alone. Olaparib was very well tolerated with only 5% rate of discontinuation for toxicity with no changes in quality of life compared to PBO as measured by the EORTC QLQ-C30 score. At this interim analysis with data maturity of only 46%, there was no OS benefit yet seen (mOS 18.9 *vs*. 18.1, HR 0.91 95% CI, 0.56–1.46, *p*=0.68). In addition to the immaturity of the data for this secondary endpoint, PARP inhibitor use in the PBO group after discontinuation of study drug and increased use of chemotherapy upon progression (49% in olaparib group *vs*. 74% in PBO group) may have contributed to the lack of OS benefit.

As shown repeatedly in both ovarian and breast cancer, the commended POLO trial has strengthened the encouragement for PARP inhibition in solid tumors, now likely setting a new standard of care in pancreatic cancer for those with germline *BRCA1* or *BRCA2* mutations. These impressive results continue to support the use of PARP inhibitors as an adjunct to DNA-damaging agents for those with homologous recombination repair deficiencies, even after prolonged exposure to platinum agents.

The growing success in this space calls for further inclusion of those with additional deficiencies of homologous recombination repair, particularly *ATM* and *PALB2* which are also of interest given their prevalence in pancreatic cancer.^18^ A phase II trial including patients with metastatic castrate resistant prostate cancer (mCRPC) treated with olaparib 400 mg twice daily identified mutations in DNA repair related genes including *BRCA 1*/*2*, *ATM*, and *PALB2* in 16 out of 49 (33%).^19^ Subgroup analysis per altered gene identified response rates defined as radiologic response per RECIST criteria or greater than 50% reduction in PSA. Response rates of 80%, 57%, and 37% were seen in patients with *BRCA 1*/*2*, *PALB2*, and *ATM* mutations respectively.^19^ The highest response rate in PSA reduction was noted in patients with *BRCA 1*/*2* and *PALB2* subgroups. This study corroborates the rationale in developing PARP inhibition in DDR-defective patients beyond *BRCA* mutations and has implications in treatment of PDAC as well.^19^ Periodic re-evaluation of those found with *BRCA* variants of uncertain significance should also be warranted for appropriate grouping of this unclear alterations to “likely benign” or “likely pathogenic” classification. The benefits of PARP inhibitor-mediated synthetic lethality for those with germline *BRCA1* or *BRCA2* mutations are now undeniable and highlights the importance of germline testing for all patients with pancreatic cancer.

## Figures and Tables

**Figure 1.**
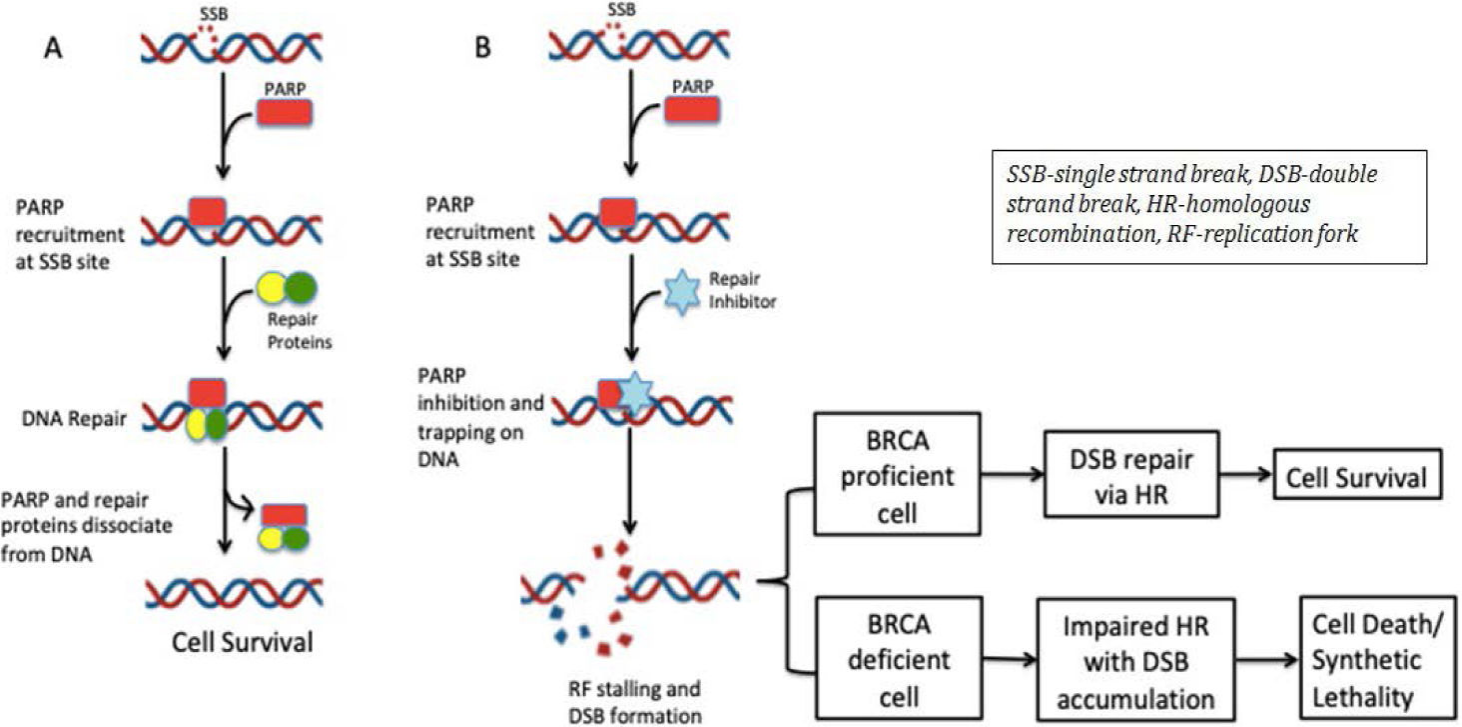
Mechanism of PARP Inhibitors Leading to Synthetic Lethality A) Normal DNA repair mechanism with functional PARP protein and DNA repair proteins B) DNA repair of SSB in the presence of PARP inhibitor resulting in DSB formation. BRCA-proficient cells have the ability to repair the DSB maintaining cell survival. BRCA deficient cells are unable to repair the accumulating double stranded breaks resulting in cell death.

**Table 1.** Current PARP Inhibitors and Food and Drug Administration (FDA) Indications

Drug	FDA Indications	Key Trials
	Ovarian Cancer	
Olaparib	Maintenance in patients with germline (gBRCAm) or somatic (sBRCAm) *BRCA* mutation with response to platinum based chemotherapy Treatment in advanced ovarian cancer with *gBRCAm* with 3 or more prior lines of chemotherapy breast cancer gBRCAm, human epidermal growth factor receptor 2 (HER2)-negative metastatic breast cancer who have previously been treated with chemotherapy in the neoadjuvant, adjuvant, or metastatic setting	SOLO-1 SOLO-2 OlympiAD
	Ovarian Cancer	
Niraparib	Maintenance treatment in patients with recurrent epithelial ovarian, fallopian tube, or primary peritoneal cancer who are in a complete or partial response to platinum-based chemotherapy	NOVA
	Ovarian Cancer	
Rucaparib	Monotherapy in patients with advanced ovarian cancer with gBRCAm or sBRCAm who have been treated with two or more lines of chemotherapy	ARIEL2

**Table 2.** POLO Trial PFS and OS

PFS (mos)	Olaparib Group	Placebo Group	Hazard Ratio	*p*-value
6	53.0%	23.0%		
12	33.7%	14.5%		
18	27.6%	9.6%		
24	22.1%	9.6%		
Median PFS	7.4-months	3.8-months	0.53	0.004
Median OS	18.9-months	18.1-months	0.91	0.68
